# Professor Emmanuel Quaye Archampong (1932–2021)

**DOI:** 10.4314/gmj.v55i4.2

**Published:** 2021-12

**Figure:**
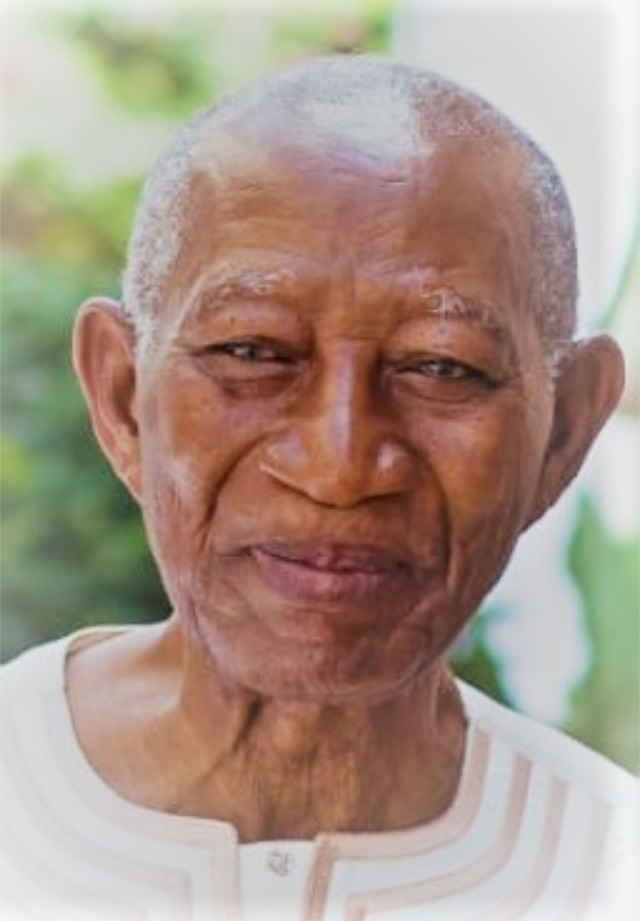


Emmanuel Quaye Archampong, Emeritus Professor of Surgery of the University of Ghana Medical School, who died in Accra on 11^th^ November 2021, was a highly regarded clinician, researcher, bedside teacher, and mentor to generations of Ghanaian and West African surgeons.

He made significant contributions to the development of undergraduate and postgraduate medical education in West Africa. He was one of three co-editors of Baja's Principles and practice of surgery, including pathology in the tropics”, which is popularly referred to as ‘BAJA’ after the initials of the editors' surname Professors EA Badoe, EQ Archampong and MOA Jaja. This textbook is a pre-eminent source of information for generations of medical undergraduates, postgraduates and surgeons, especially in the tropics. He was the Dean of the University of Ghana Medical School from 1984 to 1994 and President of the West African College of Surgeons from 1997 to 1999.

He was the Editor-in-Chief of the Ghana Medical Journal from 1973 to 1979. Before this time, he was the Assistant Editor from 1969. These years were difficult in Ghana's history, with military coups, uprisings, and social and economic challenges. This period also witnessed tension between professional groups and the military government of the day over the concept of “Union Government”, which proposed to make Ghana a non-party state. Professor Archampong managed the journal successfully and achieved regular publications. During these difficult times, he was promoted to Associate and Full Professor in 1976 and 1978, respectively. In addition, he obtained a Master of Surgery degree (M.S.) from the University of London (1974) by thesis and was awarded the Fellowship of the International College of Surgeons (USA) in 1975.

Professor Archampong excelled in his many spheres of endeavour, receiving academic, national, and international awards.

He was a recipient of the National Honours of the Republics of Ghana and Senegal. He was considered an astute administrator with a friendly posture.

Professor Archampong will be remembered as an icon in the medical profession, a great teacher, an example of dedication to duty, family, and a Christian gentleman.

Professor Archampong was laid to rest on 16^th^ December 2021 after a well-attended burial service that included the President and Vice-President of the Republic of Ghana.

May he rest in peace.

**Editorial Office**

**Ghana Medical Journal**

editor@ghanamedj.org

